# Human papillomavirus genotype distribution in Ethiopia: an updated systematic review

**DOI:** 10.1186/s12985-022-01741-1

**Published:** 2022-01-15

**Authors:** Awoke Derbie, Daniel Mekonnen, Endalkachew Nibret, Melanie Maier, Yimtubezinash Woldeamanuel, Tamrat Abebe

**Affiliations:** 1 Department of Medical Microbiology, College of Medicine and Health Sciences, Bahir Dar University, Bahir Dar, Ethiopia; 2 Centre for Innovative Drug Development and Therapeutic Trials for Africa (CDT-Africa), Addis Ababa University, Addis Ababa, Ethiopia; 3 Department of Health Biotechnology, Institute of Biotechnology, Bahir Dar University, Bahir Dar, Ethiopia; 4 College of Science, Bahir Dar University, Bahir Dar, Ethiopia; 5 Department of Diagnostics, Institute of Virology, Leipzig University Hospital, Leipzig, Germany; 6 Department of Medical Microbiology, Immunology and Parasitology, College of Health Sciences, Addis Ababa University, Addis Ababa, Ethiopia

**Keywords:** Human papillomaviruses, HPV, Genotype distribution, Ethiopia

## Abstract

**Background:**

Cervical cancer is caused by infection with high-risk human papillomaviruses (HR-HPVs). It is one of the leading causes of cancer-related deaths in Ethiopia and globally. To develop efficient vaccination and HPV-based cervical cancer screening approaches, data on genotype distribution of HPVs is crucial. Hence, the study was aimed to review HPV genotype distribution in Ethiopia.

**Methods:**

Research articles were systematically searched using comprehensive search strings from PubMed/Medline and SCOPUS. Besides, Google Scholar was searched manually for grey literature. The last search was conducted on 18 August 2021. The first two authors independently appraised the studies for scientific quality and extracted the data using Excel sheet. The pooled HPV genotype distribution was presented with descriptive statistics.

**Results:**

We have included ten studies that were reported from different parts of the country during 2005 and 2019. These studies included 3633 women presented with different kinds of cervical abnormalities, from whom 29 different HPV genotypes with a sum of 1926 sequences were reported. The proportion of high-risk, possible/probable high-risk and low-risk HPVs were at 1493 (77.5%), 182 (9.4%) and 195 (10.1%), respectively. Of the reported genotypes, the top five were HPV 16 (37.3%; 95% CI 35.2.1–39.5%), HPV 52 (6.8%; 95% CI 5.8–8.0%), HPV 35 (4.8%; 95% CI 3.9–5.8%), HPV 18 (4.4%; 95% CI 3.5–5.3%) and HPV 56 (3.9%: 95% CI 3.1–4.9%). Some of other HR-HPV groups include HPV 31 (3.8%), HPV 45 (3.5%), HPV 58 (3.1%), HPV 59(2.3%), and HPV 68 (2.3%). Among the high-risk types, the combined prevalence of HPV 16/18 was at 53.7% (95% CI 51.2–56.3%). HPV 11 (2.7%: 95% CI 2.1–3.5%), HPV 42 (2.1%: 95% CI 1.5–2.8%) and HPV 6 (2.1%: 95% CI 1.4–2.7%) were the most common low-risk HPV types.

**Conclusions:**

We noted that the proportion of HR-HPV types was higher and HPV 16 in particular, but also HPV 52, HPV 35 and HPV 18, warrant special attention in Ethiopian’s vaccination and HPV based cervical screening program. Additional data from other parts of the country where there is no previous HPV genotype report are needed to better map the national HPV genotypes distribution of Ethiopia.

**Supplementary Information:**

The online version contains supplementary material available at 10.1186/s12985-022-01741-1.

## Introduction

Cervical cancer is the most common malignancy impacting women and families globally. Today, more women are dying of cervical cancer than are dying of complications during childbirth. In 2020, an estimated 604,237 women were diagnosed with cervical cancer globally, representing 6.5% of all female cancers. In same year, the disease killed an estimated 341,843 women, 90% of whom were in less-developed regions of the world, where access to prevention, screening, and treatment services are severely limited [1]. In 2030, the annual number of new cases and death from cervical cancer has been projected to increase to 700, 000 and 400, 000, respectively [2, 3]. The global burden of cervical cancer (close to 90%) occurs in developing countries. The highest burden was reported in sub-Saharan lacking well-organized screening and Human papillomavirus (HPV) vaccination programs [2, 4–6].

Cervical cancer imposes an enormous burden in Ethiopia. The incidence and prevalence of the disease are increasing because of the growth and aging of the population, as well as an increasing prevalence of well-established risk factors [7]. According to the international agency for research on cancer assessments, the estimated number of new cervical cancer cases was 7500 in 2020 and it will be increased to 15,300 in 2040 in Ethiopia. Similarly, the mortality from the disease will increase from 5340 in 2020 to 11,000 in 2040 yearly [3]. Cervical cancer is becoming a major cause of morbidity and mortality among women in Ethiopia as the country cannot sustain proven cervical cancer prevention strategies [6].

Etiologically > 99% of cervical cancer cases are associated with genital infection with High-Risk type HPV infections. It is the most common viral infection of the reproductive tract [8, 9] that most women are experience soon after they become sexually active [10]. Persistent infection with HR-HPV is the primary cause of cervical cancer [11, 12]. More than 200 different HPVs have been characterized and completely sequenced to date. Of all types of HPVs, about 30 to 40 are sexually transmitted and infect the genital areas of both men and women [13–17]. HPVs are categorized according to their oncogenic potential. The HR-HPV types that include HPV16, 18, 31, 33, 35, 39, 45, 51, 52, 56, 58, 68 and 59. These are considered to be high-risk due to their strong implication in carcinogenesis, particularly the malignant progression of cervical tumors. Some other groups are classified as potential or probable high risk (pHR) types, including HPV 26, 53, 66, 70, 73, and 82. Studies showed that HPV 16 and 18 are the well-known oncogenic causing > 70% of all cervical cancer burdens worldwide [8, 18, 19].

The recognition of HPVs as a primary etiologic agent for human cancers has increased their medical importance and stimulated research into developing strategies for screening, diagnosis, prevention and treatment of HPV-associated diseases [20, 21]. Despite the high burden of cervical cancer related morbidity and mortality in Ethiopia, systematically compiled nationwide data on HPV genotypes distribution among Ethiopian women is limited. Therefore, this review was an update of our previous report [22] with more additional data describing the genotype distribution of HPVs in Ethiopia, where the finding would play a great role as an input for optimal vaccination and HPV-based cervical screening strategies.

## Objective

The objective of this review was to describe the genotype distribution of Human papillomaviruses among women with different kinds of cervical lesions in Ethiopia.

## Methods

### Eligibility criteria

We included observational studies that were published on peer-reviewed journals reported in English language irrespective of the year of publication and described the genotypes distribution of HPVs from cervical samples among Ethiopian women irrespective of their age group. Genotype distribution of HPVs was defined as the type of HPV identified using a molecular method from women with different cervical pathologies.

### Information sources and search strategy

This review was done following the Preferred Reporting Items for Systematic Reviews and Meta-Analysis Protocols (PRISMA) guideline [23]. Articles were systematically and compressively searched in PubMed/Medline and SCOPUS, the last search was conducted on 18 August 2021. A manual search from the Google scholar was also done for grey literature screening. The search terms were developed in line with the Medical Subject Headings (MeSH) thesaurus using a combination of key terms driven from the review objective. The first two authors (AD and DM) independently searched the articles.

The domains of the search terms were human papillomavirus, HPV, cervical cancer, cervical precancerous lesion, molecular epidemiology, genotype distribution, and Ethiopia. We combined human papillomavirus and cervical cancer/lesions with the Boolean operator “OR”, and the result was combined with the other terms with “AND”. The complete search strategy for the two databases is given in Additional file 1.

### Study selection

The research papers that have reported the genotypes distribution of HPVs among women with normal cervical cytology, cervical precancerous lesions, and cervical cancer in Ethiopian context were included. Searched articles were directly imported and handled using EndNote X9 citation manager (Thomson Reuters, New York, USA). Based on the PRISMA approach, duplicated articles were excluded, and the titles and abstracts of the remaining papers were screened independently for inclusion in full text evaluation by the first two authors (AD, DM). Differences between the authors were resolved through discussion.

### Data collection process and data items

We used an Excel spreadsheet for data extraction. The first two authors extracted the data independently. Differences between the two authors with regard to the extracted data were solved through discussion. Data such as the name of the first author, year of publication, age group of the participants, study area, the total number of women included in the study, and the type of HPVs characterized were extracted from the included articles.

### Quality appraisal

To assess the risk of bias, the two authors independently used the nine items (each score one point) based on the Joanna Briggs Institute (JBI) Critical Appraisal tools [24] for prevalence studies. Each domain had a score of 1 point. The risk of bias for each individual domain was measured as ‘yes’, ‘no’, ‘unclear’ and ‘not applicable’. In this study, ‘yes’ scored 1 and ‘no’ score zero. Therefore, the total score ranges from zero to nine, with higher scores indicating higher quality of outcome. We assumed that papers that scored > 50% (i.e. ≥ 5 of 9 scores) of the weighted value of the tool were considered as good quality. Therefore, based on our assumption, all the included articles scored above 50% positive, and we felt all good quality articles.

### Data synthesis

The data extracted from eligible studies were directly logged into a Microsoft Excel spreadsheet for synthesis and presented in terms of (1) the prevalence of HPV from each study, (2) the proportion of HR-HPVs from identified genotypes, and (3) the proportion of each identified HPV genotype. A systematic narrative synthesis was provided in which summary results were presented using text, table, and figure. Counts, ranges, and percentages were used to describe the synthesized data.

## Results

### Search results

**Fig. 1:**
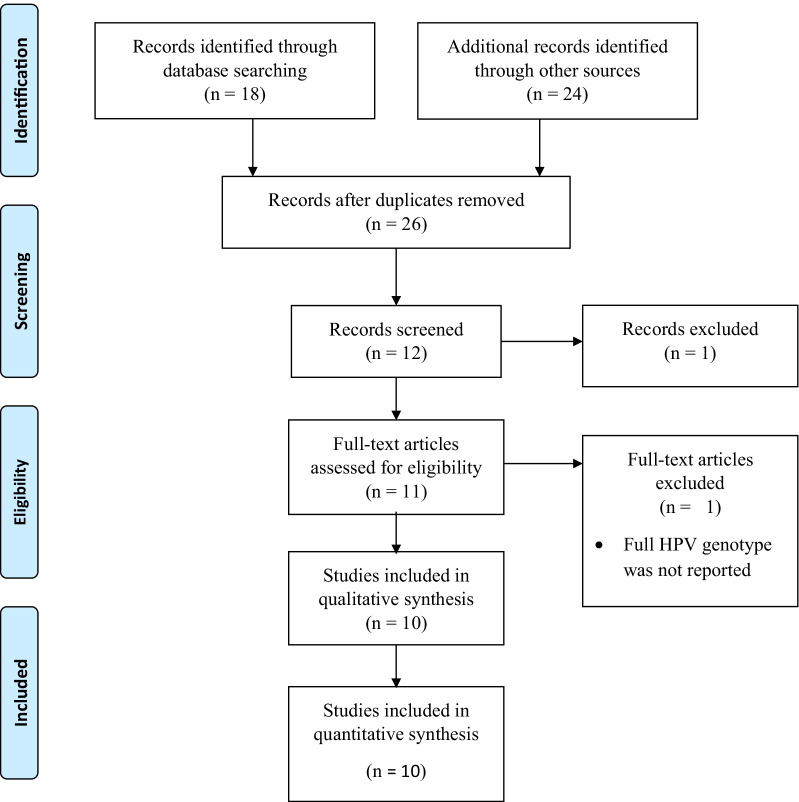
The PRISMA flow diagram of literature selection

A total of 42 articles were retrieved and screened for final inclusion. After removing the duplicate, 26 were screened by title, then 14 were removed. Consequently, 1 article was removed by abstract and 1 by full text. Finally, a total of 10 studies met our inclusion criteria. The screening was based on the PRISMA flow chart which was adapted from the PRISMA guidelines (Fig. 1).

### Study characteristics

The included studies were conducted in different parts of Ethiopia (North, Central, Southwest, and South) during 2005 and 2019. The studies recruited 3633 women (age range 15–85 years old) presented with different kinds of cervical abnormalities. Two of the studies [25, 26] used paraffin embedded cervical biopsy for HPV DNA detection and genotyping. In eight of the other studies, cervical brushes were taken from woman with different kinds of cervix presentations.

In two of the studies [27, 28], the study subjects were recruited from the general population from whom the prevalence of HPV detection was 19.9% and 23.3%. In contrast, in the rest of the articles that recruited women with different cervical abnormalities, the proportion of HPV detection was between 13.7 and 93%. The description of each study characteristic, together with the reported prevalence of HPV and the proportion of HR-HPV, is presented in Table 1.

**Table 1:** Characteristics of the included studies, 2005–2019

References	Year	Study area	Study participants	Number of study participants	Age range of the study participants	Prevalence of HPV: n (%)	Proportion of HR-HPVs: n (%)
Abate et al. [25]	2013	Addis Ababa	Paraffin embedded cervical biopsy	170	21–85	149 (93)	139 (93.5)
Bekele et al. [29]	2010	Jimma	Women with cervical dysplasia	132	32–65	82 (67.8)	82 (100)
Leyh-Bannurah et al. [27]	2014	Atta	Women who visited the gynecological outpatient department	537	15–64	107 (19.9)	86 (80.4)
Mihret et al. [30]	2014	Addis Ababa	Women having different cervical intraepithelial neoplasia	20	23–60	17 (85)	17 (100)
Ali et al. [31]	2015	Addis Ababa	Women who came for any gynecological visit	366	18–68	50 (13.7)	50 (13.7)
Fenta et al. [26]	2005	Gondar	Women who visited the gynecology outpatient dep’t	537	15–64	10(19.9)	86(16.0)
Wolday et al. [32]	2009	Addis Ababa	Women attending the out-patient clinic	233		161 (69.1)	134 (83.2)
Haile et al. [33]	2016	Adama	Women above 20 years old	83	21–65	15 (22.7)	12 (18.2)
Genbremariam et al. [34]	2016	NW* Ethiopia	Women visiting gynecology clinics	915	18–72	310 (33.9)	172 (55.5)
Teka et al. [28]	2019	Butajira	Women aged 30–49 years	893	30–49	207 (23.2)	

*NW, North-west

The included studies used PCR-based but different molecular approaches to characterize the genotype of HPVs. These include Abbott Real-Time HR-HPV PCR [31], Digene Hybrid Capture 2 HPV DNA test with genotyping Kit HPV GP [27], PCR based HPV DNA detection [26], Line probe assay (Inno-LiPA) [30], Nested PCR [32], RIATOL qPCR [33], GP5 + ⁄6+ HPV PCR with Gel electrophoresis [29], PCR using GP5 + /6 + primers [34], Reverse line blot hybridization assay [25] and multiplexed genotyping (MPG) using BSGP5 + /6 + PCR technique [28] (data not shown in Table 1).

### Genotype distribution HPVs in Ethiopia

**Fig. 2:**
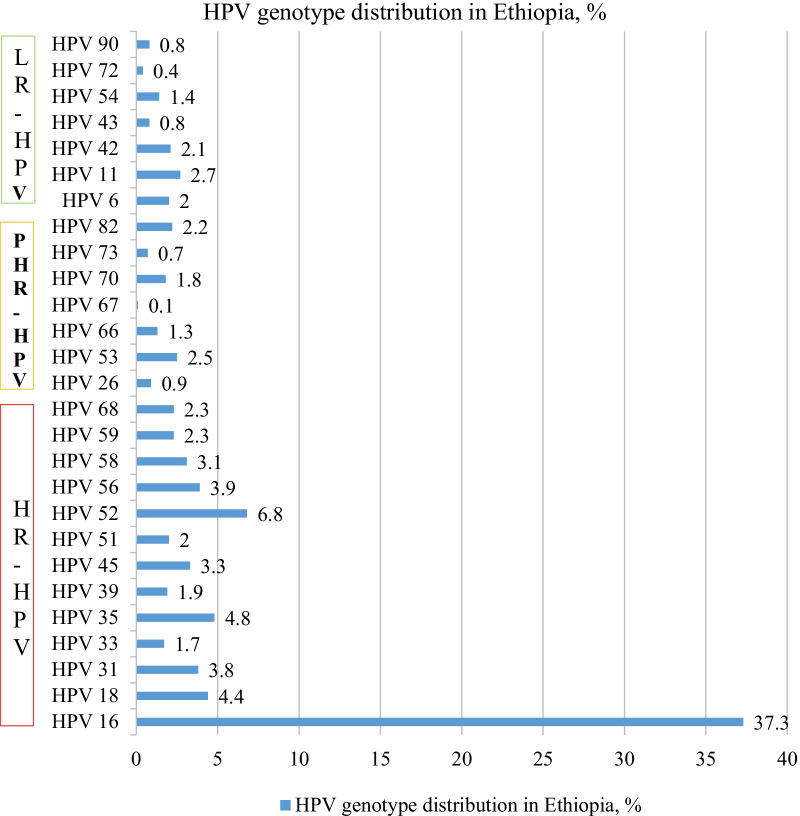
Frequency of HPV genotypes identified from the included studies, 2005–2019. LR: Low-risk, PHR: probable high-risk, HR: high-risk, HPV: Human Papillomavirus

In this review 29 different HPV genotypes with a sum of 1926 sequences were reported. The proportions of high-risk, possible/probable high-risk and low-risk HPVs were at 77.5% (1493), 9.4% (182) and 10.1% (195), respectively. Among the reported genotypes, the top five were HPV 16 (37.3%; 95% CI 35.2.1–39.5%), HPV 52 (6.8%; 95% CI 5.8–8.0%), HPV 35 (4.8%; 95% CI 3.9–5.8%), HPV 18 (4.4%; 95% CI 3.5–5.3%) and HPV 56 (3.9%: 95% CI 3.1–4.9%). Some of other reported high risk HPV groups include HPV 31 (3.8%), HPV 45 (3.5%), HPV 58 (3.1%), HPV 59 (2.3%), and HPV 68 (2.3%). Among the high-risk types, the combined prevalence of HPV 16/18 was at 53.7% (95% CI 51.2–56.3). From the low risk HPV types, HPV 11 (2.7%:95% CI 2.1–3.5%), HPV 42 (2.1%:95% CI 1.5–2.8%) and HPV 6 (2.1%:95% CI 1.4–2.7%) were predominant. The overall proportion HPV genotypes is presented in Fig. 2.

## Discussion

Because of the considerable geographical differences in the HPV genotype distribution globally, data on HPV genotyping for a specific nation is important for vaccination and support cervical cancer screening policies  to improve the effectiveness of screening programs and reduce overtreatment [35]. However, currently there is limited data on the countrywide genotype distribution of HPVs in Ethiopia.

In the present review, the detection of HPV among women who were selected from the general population was between 19.9 and 23.2%. Likewise, the prevalence of HPV from woman with different kinds of cervical dysplasia was between 13.7 and 93%. A global-based review by Forman et al. showed that the prevalence of HPV was between 11 and 12% (with higher rates, 24%, in sub-Saharan Africa) in women without cervical abnormalities [36]. The detection of HPV increases in women with cervical pathology in proportion to the severity of the lesions, which supports our finding. Forman et al., in their global-based review, also reported close to 90% detection of HPV from women with different grades of cervical abnormalities [36]. In addition, a review by Pend et al. also showed HPV prevalence to be 84.8% from different kinds of cervical lesions among Asian women [37].

In our review, the proportions of high-risk, possible/probable high-risk and low-risk HPVs were at 77.5% (1493), 9.4% (182) and 10.1% (195), respectively. A recent meta-analysis by Ogembo et al. showed that the proportion of HR-HPV was 42.2% in Eastern Africa [38]. On the contrary, a similar review that report HPV profiling from women in India, Bangladesh, Sri Lanka and Nepal reported the proportion of HR-HPV types at 97% [39]. The difference might be due to population and geographic variations. The predominantly identified genotypes were HPV 16 (37.3%), HPV 52 (6.8%), HPV 35 (4.8%), HPV 18 (4.4%) and HPV 56 (3.9%). Some of other high-risk HPV groups include HPV 31 (3.8%), HPV 45 (3.5%), HPV 58 (3.1%), HPV 59 (2.3%), and HPV 68 (2.3%). These findings are essentially necessary to predict how HPV vaccination and HPV-based screening will impact cervical cancer prevention in Ethiopia. This infers that further HPV vaccine studies in Ethiopia should mainly target the first few predominant genotypes [22].

Based on similar review reports in different parts of the globe, the genotype distribution of HPVs in different countries from different kinds of cervical lesions is presented in Table 2 for comparison.

**Table 2:** Comparison of the top five HPV genotypes with (%) distribution in different parts the world.

Our review report, HPV type (%)	Africa [38]	Asia [37]	N. America [40]	Israel [41]	Latin America and Caribbean [42]
HPV 16 (37.3)	HPV 16 (31.2)	HPV 16 (23.9)	HPV 16 (26.3)	HPV 16 (46.5)	HPV 16 (46.5)
HPV 52 (6.8)	HPV 18 (13.9)	HPV 18 (11)	HPV 31 (11.5)	HPV 18 (11.3)	HPV 18 (8.9)
HPV 35 (4.8)	HPV 31 (8.2)	HPV 58 (9.4)	HPV 51 (10.6)	HPV 31 (7.0)	HPV 31 (8.0)
HPV 18 (4.4)	HPV 33 (10.3)	HPV 56 (6.3)	HPV 53 (10.2)	HPV 51 (5.6)	HPV 58 (8.7)
HPV 56 (3.9)	HPV 35 (13.4)	HPV 52 (5.3)		HPV 45 (4.2)	HPV 33 (6.5)
Number of included studies (total number of subjects)	71 (17, 273)	14 (4198)	55 (8308)	–	79 (7986)

As it is indicated in the table, HPV 16 is the most common genotype consistently reported globally as an important cause of cervical abnormalities. Variation in the other types of HPV genotype distribution across the above studies is likely attributable to differences in population, the severity of cervical lesions, age at screening initiation, frequency, coverage, and follow-up rates of women with cervical abnormalities [22, 43]. Besides, the difference might also be associated with ethnic differences, geographical locations and the sexual behavior of their male partners [44–46].

Among HR-HPV groups HPV 16 and 18 are commonly implicated in cervical malignancies globally. In this review, the combined prevalence of HPV 16/18 was at 53.7%. Similarly, a review by Ogembo et al. showed that HPV16/18 was 45.1% from high-grade cervical lesions and 67.7% of invasive cervical cancer among African women [38]. Among HPV positive cases, the co-prevalence of HPV 16/18 was reported differently in different countries such as; 60% (in Israel) in both pre-neoplastic lesions and cervical cancer [41], 87.5% (in Central and Eastern Europe) [47], 80% (India) among high-grade cervical lesions [39]. In Italy, HPV 16 (64%) and HPV 18 (7%) were frequently reported from women with abnormal cervical cytology [48]. A review by Guan et al. revealed that HPV 16 positivity increased steeply from normal to high-grade cervical lesions [49]. Accordingly, a vaccine mixes and HPV-based screening tests should always include this genotype although some low-grade cervical lesions associated with certain other HR-HPVs may preferentially progress to cervical cancer [22, 50]. Our review would envisage the future impact of broadly identified genotypes (HPV16, 52 and 35) in vaccination and HPV-based screening in Ethiopia.

The Ethiopian Ministry of Health started vaccinating school girls aged 14 years using Gardasil-4™ (HPV 6, 11, 16, 18) since 2018.  HPV-based screening based on the detection of HPV16/18 oncoproteins, and most recently HPV DNA test were employed. This implies that vaccinating girls using Gardasil-4^TM^ and screening women for cervical lesions using HPV16/18 oncoproteins significantly reduce the number of girls who might be protected, and women who might be missed by screening, respectively as the important HPV genotypes circulating in the country are not considered very well. Most developed countries are currently using otherwise the nonavalent Gardasil®9 (6, 11, 16, 18, 31, 33, 45, 52, and 58) vaccine that targets close to 90% of all HR-HPVs [51] which is essentially an ideal type of vaccine for Ethiopians as well based our review finding. However, it might have a financial implication.

## Strength and limitation

This systematic review will be an important input to revise the current vaccination and HPV-based screening program of Ethiopia. The review includes studies from different settings and study participants, which enable us to appreciate a better picture of HPV genotypes distribution in Ethiopia. However, the review result should be interpreted in light of a few limitations. Because of the absence of studies from some parts of the country, our finding could compromise to gain  the overall picture of the HPV genotype distribution in overall Ethiopia. The other possible shortcoming of our review is the variations of HPV genotyping methods and the study participants included by the studies.

## Conclusions

In this review, HR-HPV genotypes were predominantly identified from different kinds of cervical samples. HPV 16 in particular, but also HPV 52, 35 and HPV 18, requires special attention while designing vaccination and HPV-based cervical cancer screening programs in Ethiopia. Additional data are needed to strengthen this finding and emphasize the nationwide HPV map to broadly introduce, implement and sustain effective cervical cancer prevention and control programs in the country.

## Supplementary Information

**Additional file 1**. Search strategy.

## Data Availability

All the generated data in this review are included in the manuscript. The data Excel sheet can be shared upon the request of the principal investigator.
