# Correction to: Human papillomavirus genotype distribution in Ethiopia: an updated systematic review

**DOI:** 10.1186/s12985-022-01756-8

**Published:** 2022-02-07

**Authors:** Awoke Derbie, Daniel Mekonnen, Endalkachew Nibret, Melanie Maier, Yimtubezinash Woldeamanuel, Tamrat Abebe

**Affiliations:** 1 Department of Medical Microbiology, College of Medicine and Health Sciences, Bahir Dar University, Bahir Dar, Ethiopia; 2 Centre for Innovative Drug Development and Therapeutic Trials for Africa (CDT‑Africa), Addis Ababa University, Addis Ababa, Ethiopia; 3 Department of Health Biotechnology, Institute of Biotechnology, Bahir Dar University, Bahir Dar, Ethiopia; 4 College of Science, Bahir Dar University, Bahir Dar, Ethiopia; 5 Department of Diagnostics, Institute of Virology, Leipzig University Hospital, Leipzig, Germany; 6 Department of Medical Microbiology, Immunology and Parasitology, College of Health Sciences, Addis Ababa University, Addis Ababa, Ethiopia

## Correction to: Virology Journal (2022) 19:13 10.1186/s12985-022-01741-1

Following publication of the original article [1], the authors identified an error in Fig. 2. The correct figure is given below.

**Fig. 2:**
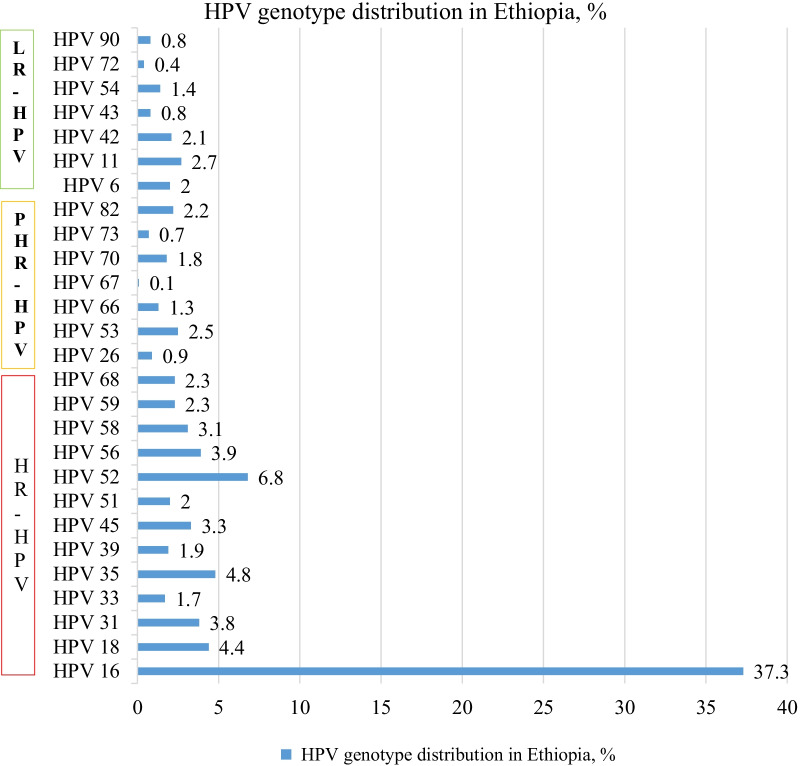
Frequency of HPV genotypes identified from the included studies, 2005–2019. *LR* low-risk, *PHR* probable high-risk, *HR* high-risk, *HPV* human papillomavirus

The original article has been corrected.
