# Supplementary material for: Human papillomavirus genotype distribution in Ethiopia: an updated systematic review
Source: Virol J. 2022 Jan 15;19:13. doi: 10.1186/s12985-022-01741-1 (PMC8760777; doi:10.1186/s12985-022-01741-1)
Supplement: Supplementary file 1 — Additional file 1. Search strategy. [file 12985_2022_1741_MOESM1_ESM.docx]

Supplement 1: Search Strategy

| PubMed | Scopus |
| --- | --- |
| ((("Molecular Epidemiology"[Mesh]) OR "Genotype"[Mesh]) AND ( "Human papillomavirus 31"[Mesh] OR "Human papillomavirus 6"[Mesh] OR "Human papillomavirus 16"[Mesh] OR "Human papillomavirus 18"[Mesh] OR "Human papillomavirus 11"[Mesh] OR "Papillomaviridae"[Mesh] OR "Papillomavirus Infections"[Mesh] OR "Alphapapillomavirus"[Mesh] )) AND "Ethiopia"[Mesh] | (TITLE-ABS-KEY (genotype) AND TITLE-ABS-KEY (distribution) OR TITLE-ABS-KEY (epidemiology) AND TITLE-ABS-KEY (“Human papillomavirus”) OR TITLE-ABS-KEY ( hpv )  AND  TITLE-ABS-KEY ( ethiopia ) ) |
